# TLR9 signaling repressed tumor suppressor miR-7 expression through up-regulation of HuR in human lung cancer cells

**DOI:** 10.1186/1475-2867-13-90

**Published:** 2013-09-03

**Authors:** Yong-Ju Li, Chun-Hong Wang, Ya Zhou, Zheng-Yuan Liao, Shun-Fei Zhu, Yan Hu, Chao Chen, Jun-Min Luo, Zhen-Ke Wen, Lin Xu

**Affiliations:** 1 Department of Immunology, Zunyi Medical College, Guizhou 563000, China; 2 Department of Chest Medicine, Qingdao Chest Hospital, Shandong 266043, China; 3 Department of Medical physics, Zunyi Medical College, Guizhou 563000, China; 4 Institute for Immunobiology and Department of Immunology, Shanghai Medical College of Fudan University, Shanghai 200032, China

**Keywords:** Toll like receptor-9, miR-7, Human antigen R, Human lung cancer cell

## Abstract

**Background:**

Our recent evidence showed that Toll like receptor 9 (TLR9) signaling could enhance the growth and metastatic potential of human lung cancer cells through repressing microRNA-7 (miR-7) expression. Human antigen R (HuR) has been involved in stabilizing multiple mRNAs in cellular biology. However, whether HuR also contributed to the altered expression of miR-7 in TLR9 signaling stimulated human lung cancer cells remains to be elucidated.

**Methods:**

The expression of HuR in human lung cancer 95D cells treated with TLR9 agonist CpG Oligonucleotides (ODNs) was detected by Real-time PCR and Western blot assay. To explore the possible role of HuR on miR-7 expression, eukaryotic expression vector encoding HuR was transiently transfected into 95D cells and then the expression of miR-7 was detected by Real-time PCR assay. Moreover, RNA interference, western blot, Real-time PCR, MTT assay, BrdU labeling, invasion assay and scratch assay were employed to examine the disrupt effect of HuR on miR-7 expression in human lung cancer cells treated with CpG ODNs. Finally, inhibitors for PI3K, Akt or Erk respectively, and western blot were performed to explore the possible signaling pathway related to HuR expression in CpG ODNs treated human lung cancer cells.

**Results:**

Our data showed that TLR9 agonist CpG ODNs could induce the expression of HuR in human lung cancer cells. Moreover, overexpression of HuR could reduce the expression of miR-7 in lung cancer cells. Notably, down-regulation of HuR using RNA interference restored miR-7 expression in CpG ODNs treated lung cancer cells, accompanied by enhanced growth and metastatic potential. Finally, CpG ODNs could induce HuR expression through Akt pathway.

**Conclusion:**

Our findings indicated that HuR could act as regulator in regulating TLR9 signaling associated biological effect in human lung cancer cells, which might be helpful for the understanding of the potential role of HuR in tumor biology.

## Background

Accumulating evidence showed that Toll-like receptors 9 (TLR9), which were mainly expressed on immune cells, were also functional expressed on lung cancer cells [1-3]. And TLR9 signaling could alter biological character of lung cancer cells including promoting the proliferation and enhancing the metastatic potential of tumor cells, indicating that activation of TLRs signaling in lung cancer cells could contribute to the progression of lung cancer [4-8]. Recent literatures further showed that miRNAs, a major class of gene expression regulators, played critical roles in regulating the biological effects of TLR9 signaling pathway on various cells. As such, miR-17-92 cluster might regulate the biological effect of CpG ODNs on chronic lymphocytic leukemia (CLL) cells [9]. One newly evidence also showed that upregulation of miRNA-574-5p was critical for TLR9 signaling enhanced tumor progression of human lung cancer [10]. However, the underlying mechanism regulating the expression of TLR9 signaling-associated miRNAs in lung cancer cells remains largely unknown.

MicroRNA-7 (miR-7), a unique member of miRNAs, played an important role in the progression of various tumors including lung cancer [11-13]. Mechanistic evidence showed that miR-7 could regulate the transduction of Akt pathway, which was critical for growth and metastasis of tumor cells [14,15]. Our most recent study also showed that downregulation of intrinsic miR-7 was important for TLR9 signaling enhanced growth and metastatic potential of human lung cancer cells [16]. However, the mechanism that downregulation of miR-7 in TLR9 signaling treated lung cancer cells remains to be investigated. Recent evidence showed that Human antigen R (HuR), a post-transcriptional regulator of gene expression, played a key role in stabilizing multiple mRNAs in cellular biology [17-19]. Interestingly, one research work further showed that HuR could regulate the expression of miR-7 in nonneural cells in brain [20]. However, whether HuR was also involved in the expression of miR-7 in TLR9 signaling treated lung cancer cells still remains to be elucidated. Here, we carefully evaluated the potential role of HuR in the expression of miR-7 on TLR9 signaling treated human lung cancer cells.

## Results and discussion

### TLR9 signaling enhanced the expression of HuR in human lung cancer cells

**Figure 1:**
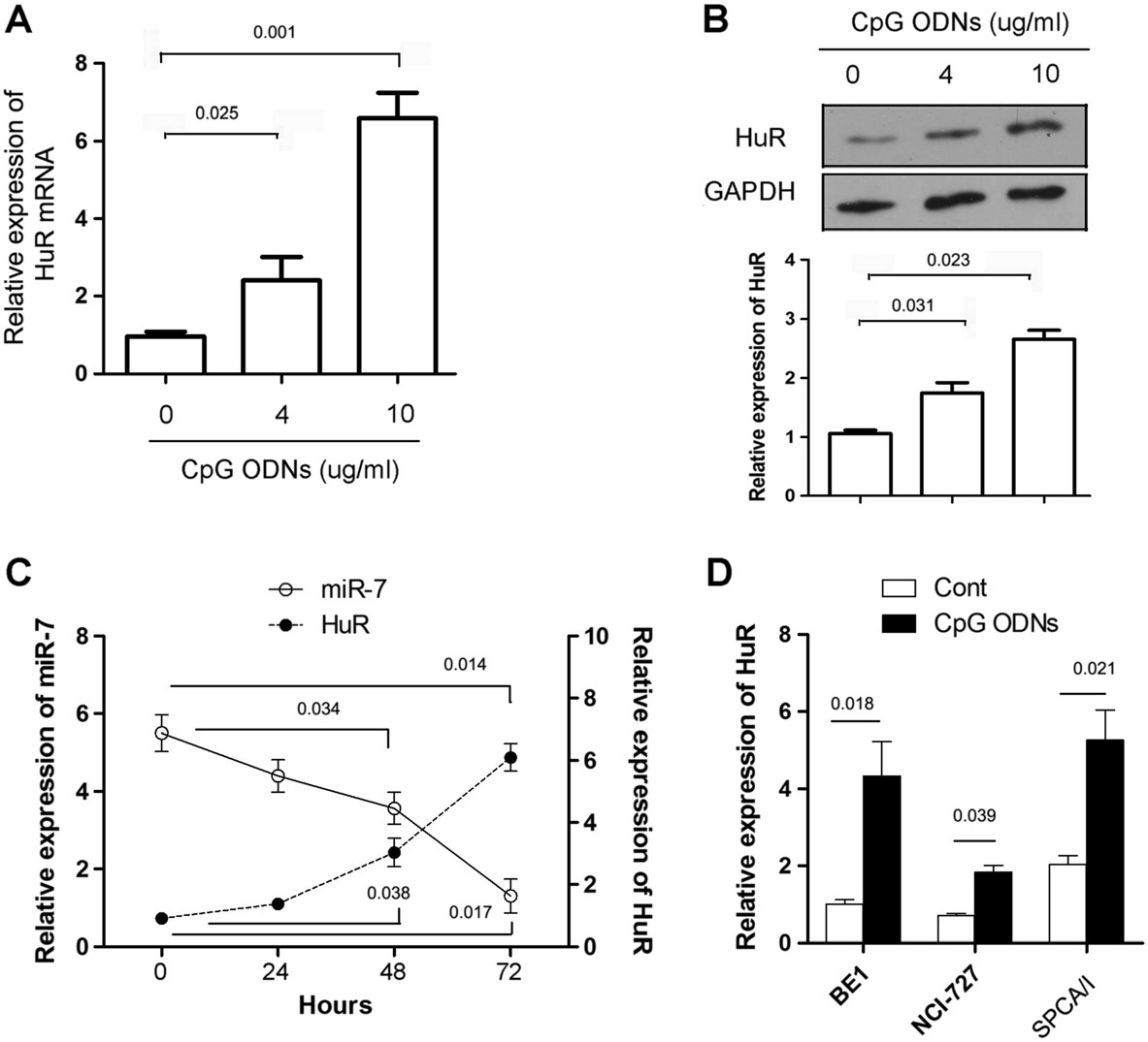
**TLR9 signaling enhanced the expression of HuR in human lung cancer cells. (A)** 95D cells were treated with indicated dose of CpG ODNs. 72 hrs later, the expression level of HuR mRNA was detected by Realtime PCR assay. **(B)** The expression of HuR protein also was detected by Western Blot and calculated. **(C)**. 95D cells were treated with 10 μg/ml CpG ODNs. Then, the expression of miR-7 and HuR were analyzed at indicated time point. **(D)** Human lung cancer cell line BE1, NCI-H727 and SPCA/I cells were treated with 10 μg/ml CpG-ODNs respectively for 72 hrs. Then the expression level of HuR mRNA was detected by Realtime-PCR assay. One representative data of three independent experiments was shown.

To investigate the potential role of HuR on the expression of miR-7, we firstly detected the expression of HuR in CpG ODNs, TLR9 agonist, treated human lung cancer cells. As shown in Figure 1A and B, we found that CpG ODNs could significantly enhance the expression of HuR mRNA and protein in human lung cancer cell line 95D cells in a dose dependent manner (p < 0.05). Next, we further detected the expression of miR-7 on 95D cells. As shown in Figure 1C, the expression of miR-7 decreased during the stimulation of CpG ODNs, accompanied by elevated expression of HuR (p < 0.05), which was consistent with our previous data [16].

Our previous study showed that CpG ODNs could also reduce miR-7 expression in other lung cancer cells such as BE1, NCI-H727 and SPCA/I [16], which also expressed TLR9 molecule (data not shown). Then, to confirm above phenomenon, we observed the expression level of HuR in lung cancer cell line BE1, NCI-H727 and SPCA/I cells. Consistently, we found that CpG ODNs also obviously elevated the expression level of HuR in BE1, NCI-H727 and SPCA/I respectively (Figure 1D, *p* < 0.05). These data strongly suggested that TLR9 signaling could significantly enhance the expression of HuR in lung cancer cells.

### Overexpression of HuR reduced the expression of miR-7 in human lung cancer cells

**Figure 2:**
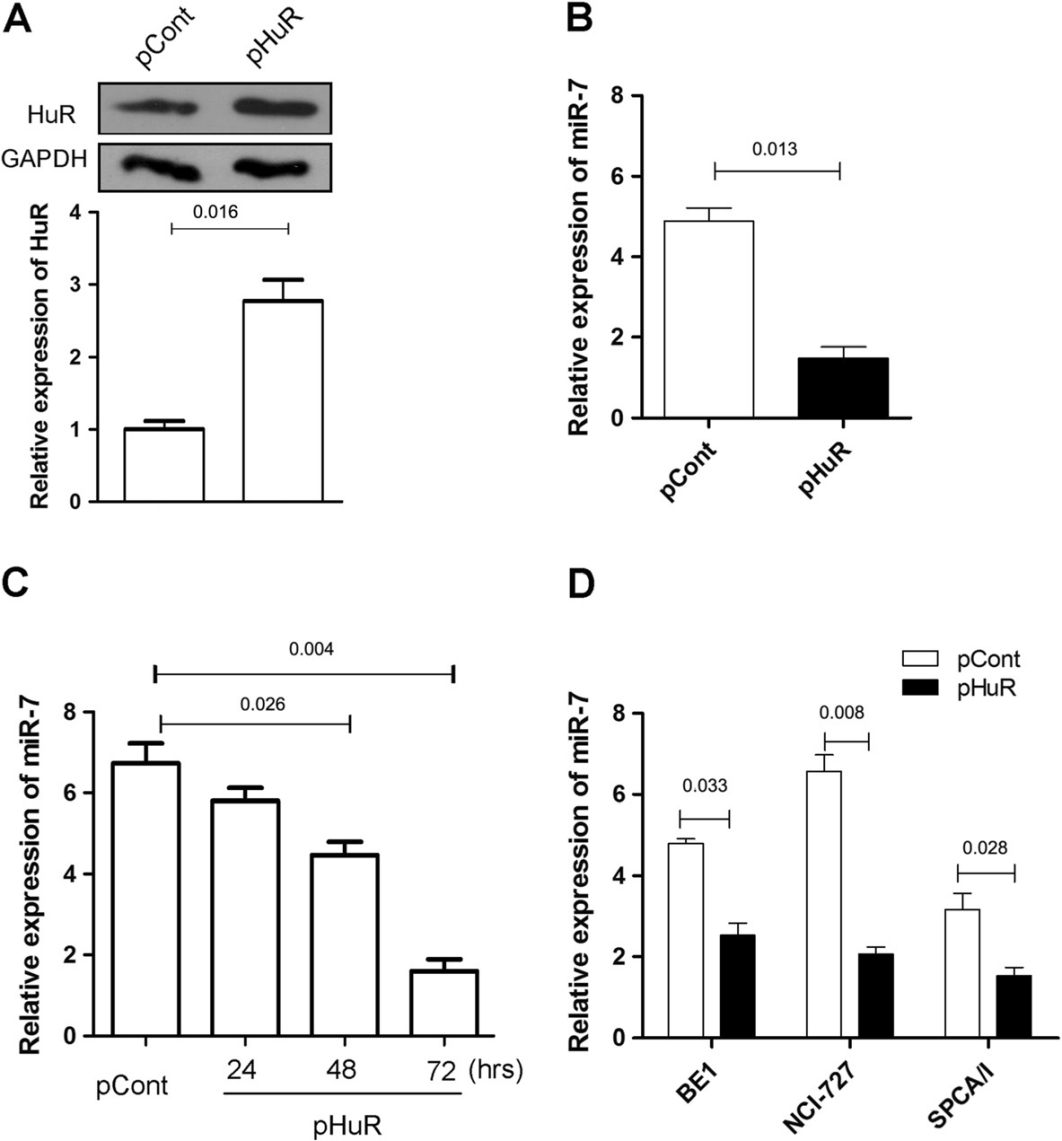
**Overexpression of HuR reduced the expression of miR-7 in human lung cancer cells.** 5 × 10^4^ 95D cells were transiently transfected with plasmid p-HuR (100 μg) or p-Cont (100 μg) and cultured in 24-well plate. **(A)** 72 hrs later, the expression of HuR was analyzed by Western Blot and calculated. **(B)** The relative expression of miR-7 was determined by Realtime PCR assay. **(C)** 95D cells were transiently transfected with plasmid p-HuR (100 μg) or p-Cont (100 μg). The relative expression of miR-7 was further determined by Real-time PCR assay at indicated time point. **(D)** Human lung cancer cell line BE1, NCI-H727 and SPCA/I cells were also transiently transfected with 100 μg plasmid p-HuR or p-Cont and cultured in 24-well plate respectively. 72 hrs later, the relative expression of miR-7 also was determined by Real-time PCR assay. One representative data of three independent experiments was shown.

Next, we further investigated whether HuR could regulate the expression of miR-7 in human lung cancer cells. We constructed and transiently transfected the eukaryotic expression vector encoding HuR (termed as pHuR) into human lung cancer cells. Our data showed that expression level of HuR in pHuR transfected group was higher than that in control group (Figure 2A, p < 0.05). Importantly, we found that the expression of miR-7 decreased obviously in pHuR transfected group in a time dependent manner (Figure 2B and C, p < 0.05).

To validate these finding, we further observed the effect of HuR overexpression on the expression of miR-7 in other lung cancer cells. Similarly, the expression level of miR-7 in pHuR transfected human lung cancer cells BE1, NCI-H727 and SPCA/I also decreased respectively (Figure 2D, p < 0.05). These data demonstrated that HuR could regulate the expression of miR7 in human lung cancer cells.

### Down-regulation of HuR elevated the expression of miR-7 in CpG ODNs treated human lung cancer cells

**Figure 3:**
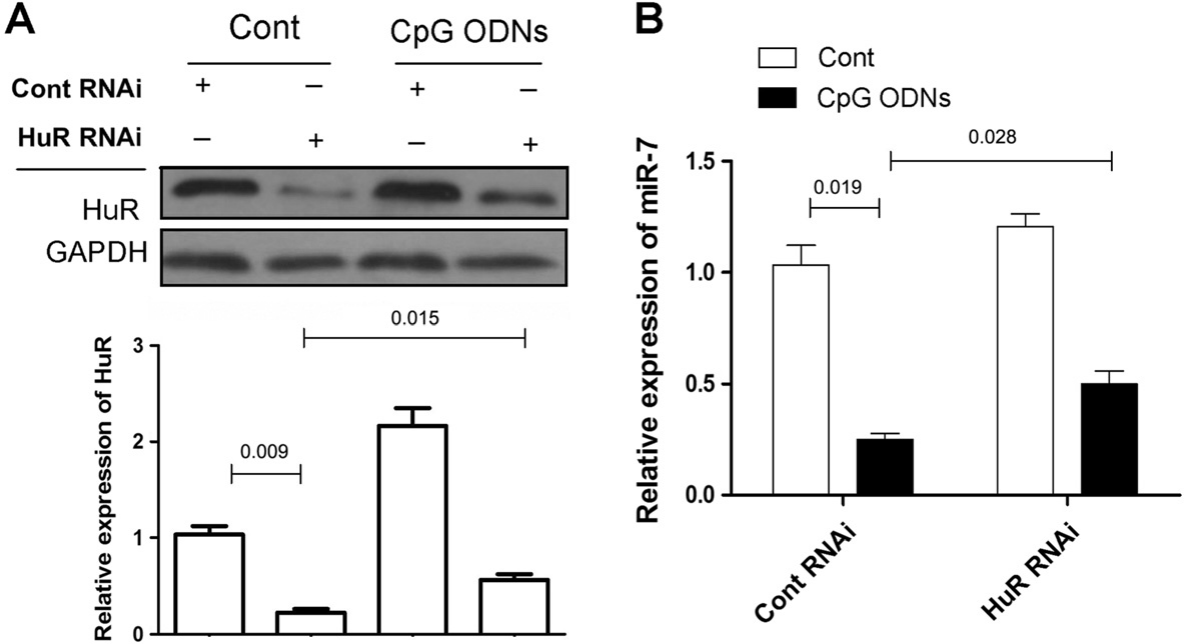
**Down-regulation of HuR reversed the expression of miR-7 in CpG ODNs treated human lung cancer cells.** 95D cells were transiently transfected with HuR RNAi (10 nmol) or control RNAi (10 nmol) respectively and then treated with CpG ODNs (10 μg/ml). 72 hrs later, the expression of HuR was determined by western blot and calculated **(A)**. **(B)** The relative expression level of miR-7 was detected by Real-time PCR assay. One representative data of three independent experiments was shown.

To determine whether up-regulation of HuR contributed to TLR9 signaling induced repression of miR-7, we downregulated HuR expression using RNAi and then detected the expression of miR-7 in human lung cancer cells. As shown in Figure 3A, HuR RNAi could significantly reduce the expression of HuR in CpG ODNs treated 95D cells (p < 0.05). Importantly, we found that the expression level of miR-7 in HuR RNAi transfected group treated with CpG ODNs was significantly higher than that in control group (Figure 3B, p < 0.05), indicating that downregulation of HuR could reverse the expression of miR-7 in human lung cancer cells.

### Down-regulation of HuR abrogated TLR9 signaling enhanced growth and metastatic potential of human lung cancer cells

**Figure 4:**
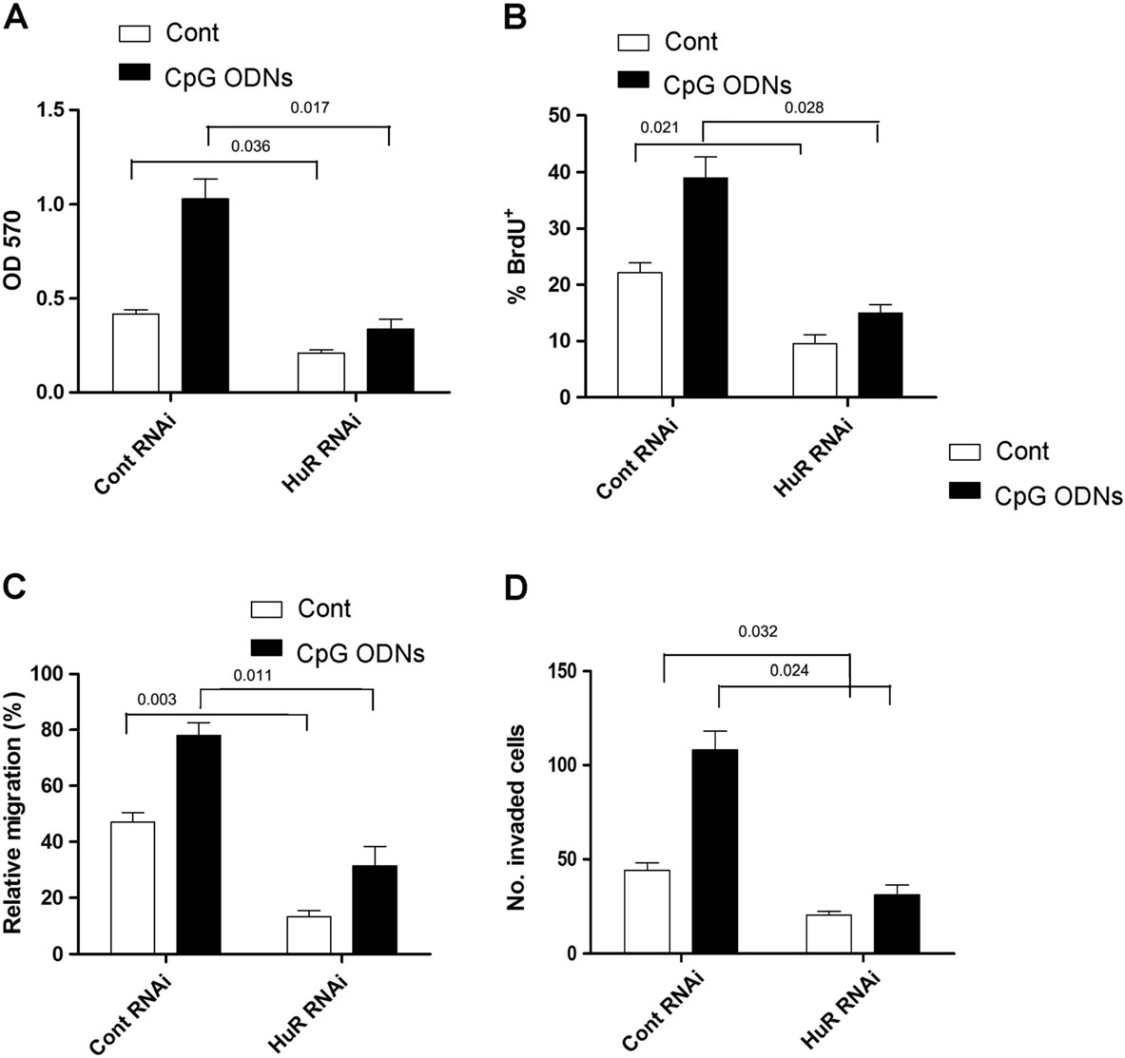
**Down-regulation of HuR reduced TLR9 signaling enhanced growth and metastatic potential of human lung cancer cells.** 95D cells transiently transfected with HuR RNAi (10 nmol) or control RNAi (10 nmol) were cultured in the presence of 10 μg/ml CpG ODNs. 72 hrs later, the proliferation of cells also was determined by MTT assay **(A)** and BrdU incorporation assay **(B)**. **(C)** The migration of 95D cells in each group was performed by transwell assay. **(D)** The invasion ability of 95D cells in each group also was determined by invasion assay. One representative data of three independent experiments was shown.

Our previous data showed that TLR9 signaling could enhance the growth and metastatic potential of human lung cancer cells through altering miR-7 expression [7,16]. Then, we further investigated whether up-regulation of HuR was involved in the effect of TLR9 signaling on human lung cancer cells. As shown in Figure 4A and B, we found that CpG ODNs stimulation could effectively increase the growth of in 95D cells in vitro, which was consistent with our previous work [7]. Importantly, we found that TLR9 signaling enhanced growth of 95D cells was significantly reduced in HuR RNAi transfected group in vitro (Figure 4A and B, *p* < 0.05), indicating down-regulation of HuR could reduce TLR9 signaling enhanced growth of human lung cancer cells.

Next, we further investigated whether down-regulation of HuR could also influence the metastatic potential of 95D cells enhanced by TLR9 signaling. As shown in Figure 4C and D, TLR9 signaling enhanced migration and invasion capacity of 95D cells in vitro was also significantly reduced in HuR RNAi transfected group (*p* < 0.05). Combining these data suggested that up-regulation of HuR was be involved in TLR9 signaling enhanced growth and metastatic potential of human lung cancer cells.

### TLR9 signaling enhanced the expression of HuR through Akt pathway in human lung cancer cells

**Figure 5:**
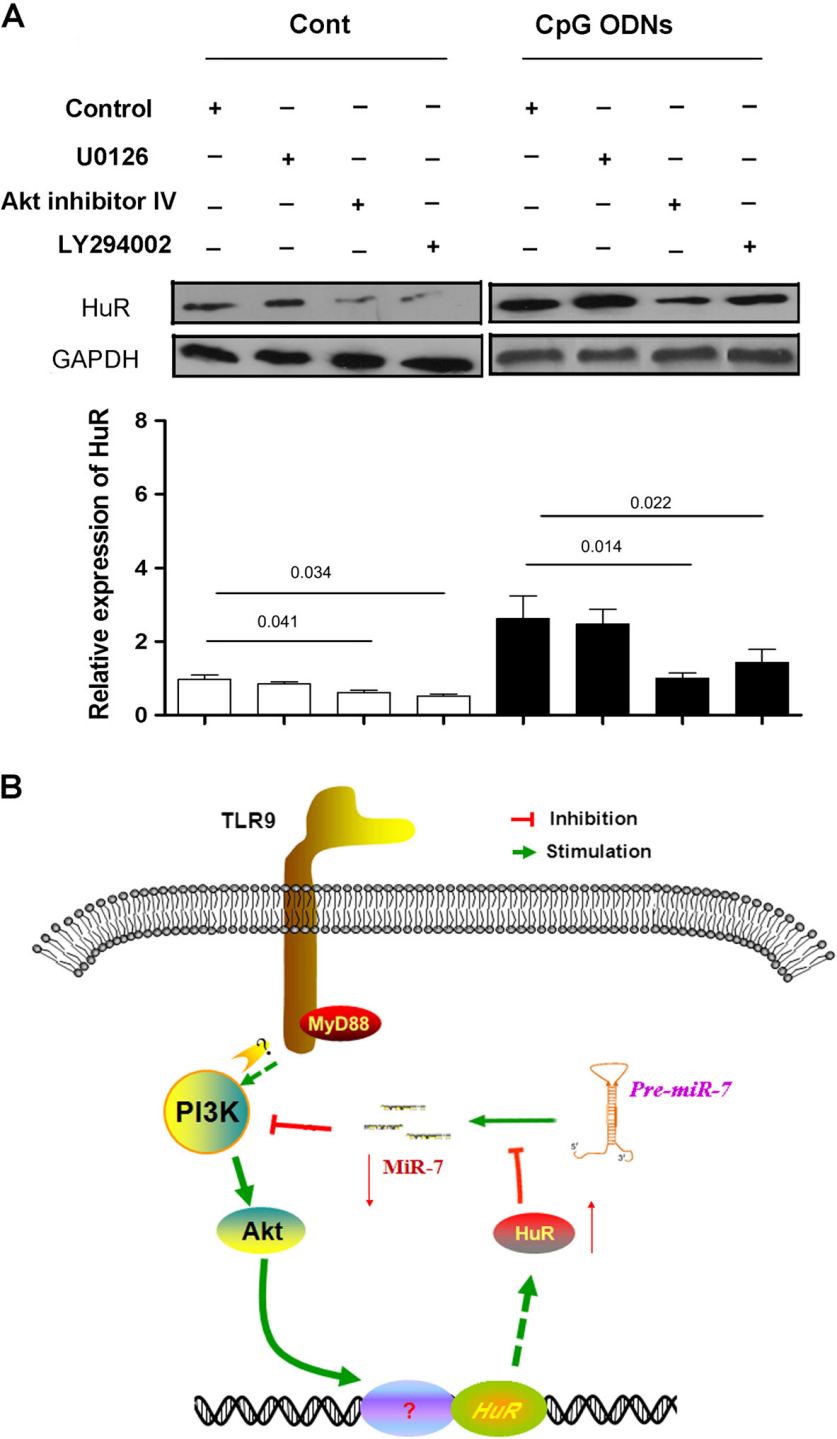
**TLR9 signaling induced the expression of HuR through Akt pathway.** 95D cells were treated with 50 μM PI3K inhibitor (LY294002) or specific MEK (Mitogen-activated protein kinase) inhibitor (U0126), 2.5 μM Akt inhibitor IV respectively, and then cultured in 24-well plate in the presence of 10 ug/ml CpG ODNs. 72 hrs later, the expression of HuR protein was determined by western blot and calculated **(A)**. **(B)** Schematic diagram for TLR9 signaling induced expression of HuR.

Previous works showed that PI3K pathway inhibitor could alter the expression of HuR in human hepatoma cell line, suggesting PI3K/Akt pathway was important for HuR expression [21]. To reach a comprehensive understanding, we further treated 95D cell with PI3K inhibitor (LY294002) and specific MEK (Mitogen-activated protein kinase) inhibitor (U0126). As shown in Figure 5A, Akt inhibitor completely blocked TLR9 signaling induced expression of HuR (p < 0.05). However, the expression of HuR in U0126 treated group did not change significantly (p > 0.05), indicating ERK1/2 did not involved in TLR9 signaling induced HuR expression in lung cancer cells.

To further confirm the role of PI3K/Akt pathway in TLR9 signaling induced HuR expression, we next treated 95D cells with Akt inhibitor. Consistently, Akt inhibitor also could reduce the expression of HuR induced by CpG ODNs (Figure 5A, p < 0.05). In addition, the expression level of miR-7 also increased significantly (data not shown), which was consistent with our previous work [16]. Combing these data suggested that PI3K/Akt pathway was critical for TLR9 signaling induced expression of HuR in human lung cancer cells.

## Discussion

Accumulating evidence showed that HuR was expressed in various tumor cells and played an important role in the biology of various tumor cells through post-transcriptionally regulating the stabilization of multiple AU-rich element-bearing mRNAs [17,22,23]. Such as, Kurosu et al. reported that HuR could keep an angiogenic switch on by stabilising mRNA of VEGF and COX-2 in tumor endothelium [18]. Moreover, Blaxall et al. found that the expression of HuR was important for the maintenance and progression of tumor cells in neoplastic lung tissue [24]. Recently, Kim et al. further reported that HuR was highly expressed on clinical lung cancer tissues and stabilizes the expression of cyclooxygenase-2 (Cox2) [25]. Our present work extended these previous works by demonstrating that TLR9 signaling could enhance the expression of HuR. Importantly, we further found that up-regulation of HuR was contributed to TLR9 signaling enhanced growth and metastatic potential of human lung cancer cells. These finding might support the fact that HuR could be an important intrinsic regulator in distinct tumor cells, which ultimately contributed to tumor biology.

Recently, miR-7 was reported played an important role in regulating the biology of various tumor cells through repressing the expression of different target molecules. In previous study, we reported down-regulation of intrinsic miR-7 was critical for TLR9 signaling enhanced progression of human lung cancer cells through altering the expression of PIK3R3 [16]. As a tumor suppressor, the expression of miR-7 was commonly repressed in tumor cells. Such as, Kong et al. reported that activated macrophage-derived small molecule could reduce the expression of miR-7 in gastric tumor cells [26]. Reddy et al. reported that homeodomain transcription factor (HoxD10) could regulate the expression of miR-7 through binding to the promoter site of miR-7 in breast cancer cells [27]. Our current work further reported that HuR could regulate the expression of miR-7 in human lung cancer cells. Consistently, Choudhury et al. found that HuR could bind to the conserved terminal loop of pri-miR-7 and regulate the expression of miR-7 in nonneural cells in brain tissue [21]. In addition, it should be noted that our previous data also showed the activity of miR-7 promoter also decreased in TLR9 signaling treated human lung cancer cells [16]. Combining these data suggested that the underlying mechanism regulating expression of distinct miRNAs such as miR-7 in different cells was distinct and complex, which related to different transcriptional and post-transcriptional mechanisms. Therefore, the related transcriptional mechanism still remains to be further elucidated.

Some literatures showed that the expression of HuR was regulated through transcriptional and post-transcriptional mechanisms [28,29]. For example, Mansfield et al. reported that Neuron-specific ELAV/Hu proteins suppress HuR mRNA during neuronal differentiation by alternative polyadenylation [30]. Dai et al. further found that HuR could autoregulate its expression by promoting alternative polyadenylation site usage [31]. However, the possible signaling pathway involved in regulation on HuR expression remains largely unknown. It was well documented that PI3K/Akt pathway was a critical for tumor biology. Our previous study also showed that PI3KAkt pathway was critical for TLR9 signaling enhanced metastatic potential of lung cancer cells [16]. In present study, we further demonstrated that TLR9 signaling could enhance the expression of HuR through Akt pathway, which ultimately reduce the expression of miR-7, suggesting that PI3K/Akt pathway was important for the expression of HuR in cancer cells. Similarly, one most newly work also reported that Akt signaling could enhance the expression of HuR, which binded to Grb10 and inhibits apoptosis of renal proximal tubule cells by amplifying Akt signaling through a positive feedback loop [21]. In additions, we also found that overexpression of miR-7 could significantly reduce the expression of HuR in CpG ODNs treated human lung cancer cells (Additional file 1: Figure S1), through inhibiting the transduction of PI3K/Akt pathway [16]. Therefore, as shown in Figure 5B, we presumed that during the treatment of TLR9 agonist CpG ODNs on human lung cancer cells, TLR9 signaling induced the expression of HuR via PIK3/Akt pathway. Up-regulated HuR could bind to the loop sites of pri-miR-7 and reduce the expression of miR-7 [20], thereby synergizing the transduction of PI3K/Akt pathway as a positive feedback loop, which ultimately resulted in enhanced growth and metastatic potential of human lung cancer cells.

## Conclusions

To our knowledge, it is the first time TLR9 signaling was identified could enhance the expression of HuR in human lung cancer cells. Importantly, in contrast to previous findings, we characterized that up-regulation of HuR was contributed to TLR9 signaling enhanced growth and metastatic potential of human lung cancer through altering the expression of miR-7. Our findings indicated that HuR could act as regulator in regulating TLR9 signaling associated biological effect in human lung cancer cells through a positive feedback loop, which might be helpful for the understanding of the potential role of HuR in tumor biology.

## Materials and methods

### Reagents and cell line

The following oligonucleotides (ODNs) were used and purchased from Integrated DNA Technologies (Coralville, IO): CpG ODNs 2216 5′-GGGGGACGATCGTCGGGGG-3′; control, ODNs1612: 5′-GCTAGAGCTTAGGCT-3′. Human lung cancer cell line 95D cells, NCI-H727 cells, BE1 cells and SPCA/I cells were cultured at 37°C under 5% CO_2_ in completed RPMI 1640 (GIBICO, Grand land, NY, USA) medium. HuR RNAi and corresponding control RNAi were purchased from Novus Biologicals (No.H00001994-R01). Akt inhibitor IV, PI3K inhibitor (LY294002) and specific MEK (Mitogen-activated protein kinase) inhibitor (U0126) was purchased from Merck. All other reagents were purchased from Sigma-Aldrich unless stated otherwise.

### Real-time PCR assay

Total cellular RNA and cDNA were prepared as previously described [16]. HuR levels were measured by SYBR Green-based Realtime PCR using Light Cycler (Roche, USA). Reverse transcriptase reactions and real-time PCR were performed according to the manufacturer’s protocols. The sequences were as follows: HuR primers: 5′- CCTGTTCAGCAGCATTG-3′ and 5′- GGCGAGCATACGACAC-3′. Cycle threshold (CT) values were compared against a standard curve to estimate starting amounts of mRNA, and the relative expression of HuR mRNA was estimated by normalizing these values against 18S rRNA CT values were generated using a preoptimized 18S rRNA primer set (Applied Biosystems, FosterCity, CA). The relative expression of miR-7 was performed according to our previous description [16].

### Plasmid construction and preparation

The gene for the HuR (NM_001419.2) were expanded by RT-PCR from human mRNA derived from 95D cells using forward primer (5′- CGGAATTCAATACAATGTCTAATGGTTATG-3′) and a reverse primer (5′-GGGGTACCATTGGCGCAAAATGAG-3′) and then subcloned into *EcoR I* and *Kpn I* sites of pcDNA3.1 vector (Invitrogen Corp., San Diego, California, USA) to generate pcDNA3.1-HuR plasmid (termed as pHuR). Clone identity was verified using restriction digest analysis and plasmid DNA sequencing. Endotoxin-free plasmids were obtained using Endofree plasmid mega kit (QIAGEN GmbH, Hilden, Germany). Then, plasmids were transiently transferred into the 95D cells using Lipofectamine-2000 (Invitrogen) in different following experiments according to the manufacturer’s instruction.

### Cell proliferation assays

95D cells transiently transfected with 10 nmol HuR RNAi or Scramble control using Lipofectamine-2000 (Invitrogen) were seeded at 3 × 10^3^ cells each well and incubated in the presence of 10 μg/ml CpG ODNs at 37°C in 5% CO_2_ in 96 well plates for 72 hrs. Assessment of cell proliferation was measured in terms of optical absorbance (OD) per well by a semi-automated tetrazolium-based colorimetric assay using MTT.

### BrdU labeling

95D cells transiently transfected with 10 nmol HuR RNAi or Scramble control were treated with CpG ODNs as described in the previous report [7]. After 48 hrs, final concentration of 5 mmol/ml BrdU (5-bromo-2-deoxyuridine; Sigma) was added. 4 hrs later, 95D cells were collected and the proliferation was analyzed by FACS.

### Invasion assay

The invasive assay was done as described previously [16]. 95D cells transiently transfected with 10 nmol HuR RNAi or control RNAi were placed in the upper wells in the presence of 10 μg/ml CpG ODNs or control ODNs and the lower wells were filled with fibroblast-conditioned medium. After incubation for 24 hrs, cells on the lower surface of the membrane were stained by the H&E method and counted under a light microscope (×200).

### Western blotting

Western blotting was performed on cytosolic cellular extracts as described previously [16]. The membrane was washed in 5% skim milk in phosphate buffered saline + 0.03% Tween 20 (PBS-T) for 1 hrs in order to block nonspecific protein binding sites on the membrane. Immunoblotting was performed using a monoclonal antibody to HuR (Santa Cruz, SC-5261) at a dilution of 1/1000 in a nonfat milk-Tris buffer. The membrane was then washed and subsequently probed with a corresponding secondary anti-mouse antibody conjugated to horseradish peroxidase (Amersham Life Sciences) at a dilution of 1:5000 and developed with chemiluminescence (Pierce, IL). The membrane was then exposed to X-ray film (Kodak, NY) which was subsequently developed.

### Statistical analyses

Statistical analyses of the data were performed with the aid of analysis programs in SPSS12.0 software. Statistical evaluation was performed using one-way analysis of variance (ANOVA; *p* < 0.05) using the program PRISM 5.0 (GraphPad Software Inc., San Diego, CA, USA).

## Abbreviations

TLRs: Toll like receptors; ODNs: Oligonucleotides; miR-7: microRNA-7; RNAi: RNA interference; HuR: Human antigen R.

## Competing interests

The authors declare that they have no competing interests.

## Authors’ contributions

LYJ and WCH executed the western blotting, Realtime-PCR experiments and drafted the manuscript. ZY and LZY both participated in the RNAi experimental procedures. HY and CC performed MTT and Invasion assay. ZSF and LJM performed inhibition assay and BrdU labeling assay. WZK contributed to the design of the study, the analysis of the data, and drafted the manuscript. XL supervised the project, making substantial contributions to the concept and design of the study, analyzing and interpreting the data, and writing the manuscript. All authors read and approved the final manuscript.

## Supplementary Material

Additional file 1: Figure S1 Overexpression of miR-7 reduced HuR expression in human lung cancer cells treated with CpG ODNs. Click here for file
