# Supplementary material for: TLR9 signaling repressed tumor suppressor miR-7 expression through up-regulation of HuR in human lung cancer cells
Source: Cancer Cell Int. 2013 Sep 3;13:90. doi: 10.1186/1475-2867-13-90 (PMC3847485; doi:10.1186/1475-2867-13-90)
Supplement: Additional file 1: Figure S1 — Overexpression of miR-7 reduced HuR expression in human lung cancer cells treated with CpG ODNs. [file 1475-2867-13-90-S1.docx]

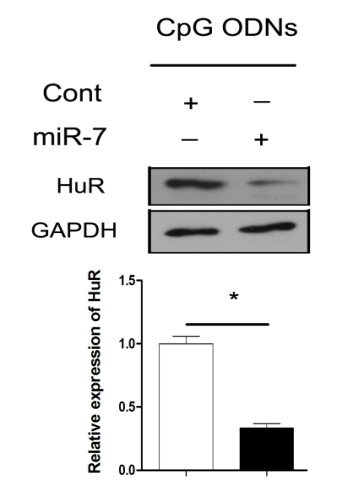


**Additional fig 1. Overexpression of miR-7 reduced HuR expression in human lung cancer cells treated with CpG ODNs.**

95D cells transiently transfected with miR-7 mimics (10nM) or Scramble control (10nM) were stimulated by CpG ODNs for 48 hrs. The expression level of HuR on 95D cells were analyzed by Western Blot and calculated. *P<0.05
